# European Board for Accreditation in Cardiology (EBAC) 2015 CME/CPD Survey Summary

**DOI:** 10.3402/jecme.v5.29757

**Published:** 2016-01-07

**Authors:** Julie Simper, Robert Schaefer, Reinhard Griebenow, Lampros Michalis, Heinz Weber

**Affiliations:** 1 International CME/CPD Consulting, Amsterdam, The Netherlands; 2 CEO EBAC, Cologne, Germany; 3 Chairman, EBAC Advisory Committee, EBAC, Cologne, Germany; 4 Vice-President Education, UEMS Cardiology Section, Brussels, Belgium; 5 Chairman, Foundation Council, European Cardiology Section Foundation (ECSF), Cologne, Germany

**Keywords:** Survey, CME/CPD, provider, EBAC, accreditation, needs assessment, conflict of interest, evaluation, respondents

## Abstract

In the spring of 2015, the European Board for Accreditation in Cardiology (EBAC) collaborated with International CME/CPD Consulting to design and administer a survey to approximately 1,171 professionals active in the field of European CME/CPD, with a focus on cardiology. With a nearly 5% response rate, the results herewith are non-representative, but do express current behaviours and attitudes of those active in European accredited CME/CPD.

## Commonly Used Acronyms

CME – Continuing Medical Education

CPD – Continuing Professional Development

EBAC – European Board for Accreditation in Cardiology

EFPIA – European Federation of Pharmaceutical Industries and Associations

UEMS – European Union of Medical Specialists 

EACCME – European Accreditation Council for Continuing Medical Education

NA – Not Applicable

PCO – Professional Congress/Conference Organiser

RCP – Royal College of Physicians

## Introduction

The European Board for Accreditation in Cardiology (EBAC) is accountable for the accreditation of international CME activities in cardiology for the European medical community. Beyond accrediting CME activities, EBAC takes responsibility for the development, availability, and quality of international CME in European cardiology. Practically, this means that EBAC also:

- **Advises** and helps those who wish to establish accreditation procedures in cardiology
- **Facilitates** individual cardiologist's international CME credit acquisition
- **Encourages** the recognition of those credits by national authorities
- **Produces guidelines** for CME in cardiology

With this broader mission in mind, EBAC reached out to the European cardiology CME/CPD provider community in the spring of 2015 to survey those implementing the accreditation criteria and submitting applications. The goal was to validate certain observations made by EBAC reviewers and obtain some indications on trends and opinions in the quickly changing environment that is European CME/CPD. The insight gained would be used to help EBAC improve and facilitate its accreditation process. This report represents a summary of the survey process and overall results.

## Overview

EBAC engaged the assistance of Julie Simper from International CME/CPD Consulting, an independent CME/CPD professional with over 13 years’ experience internationally. Creation of the questionnaire was a collaborative effort between the two organisations, whereas the survey administration and presentations of results were solely managed by International CME/CPD Consulting. In this way, all responses remained anonymous, with only summarised and anonymised information provided to EBAC.

The survey questions originated from several initial considerations including EBAC accreditation applicant demographics and data on those accreditation requirements most commonly done incorrectly or posing the most difficulty for applicants. The questionnaire had a total of 29 questions structured into four categories.

1. **General*** (questions 1–9)*
  High-level questions to assess the respondent profile: organisation type, years in CME/CPD for both the organisation and individual, types of activities organised, geographic reach, and perceived value of accreditation.
2. **Implementing the Accreditation Criteria*** (questions 10–18)*
  Considering the various accreditation criteria and rules in general and how respondents integrate them into operations. For example, the role of physician leads versus the organisational team in activity planning (in theory and in practice), the ability to understand and apply the various criteria areas, topic selection, and evaluation practices.
3. **EBAC Accreditation and Support*** (questions 19–24)*
  Questions related specifically to respondents’ experience with EBAC: number of applications annually, satisfaction with various elements of the EBAC accreditation process, suggestions to accreditors, desired changes to the accreditation process, and miscellaneous questions/comments.
4. **EBAC Provider and Sponsor Meeting*** (questions 25–29)*
  EBAC hosted an educational meeting for CME/CPD providers and sponsors in May 2015. The survey included several questions addressing intentions to attend and topics/questions respondents wished to see addressed. Responses to these questions are not included in this report, but can be downloaded with the full survey results.

The questionnaire was tested on several providers generously donating their time and constructive feedback, resulting in final adjustments to improve flow, comprehension, and limitation to approximately 15 min to complete. The survey was designed in Survey Monkey for online administration and co-branded by EBAC and International CME/CPD Consulting. It was open for 3 weeks: March 17–April 7, 2015. Three email invitations were sent from International CME/CPD Consulting to the complete EBAC database of those having interactions with EBAC to obtain European accreditation in cardiology. The database consisted initially of 1,449 contacts; however, 278 email addresses were undeliverable for a multitude of reasons. Therefore, 1,171 contacts were presumed to have received the survey invitation. Participation in the survey was on a voluntary basis; respondents were not incentivised or compensated for taking the survey, nor were responses linked to any EBAC applications.

## Results and discussion

Fifty-five respondents completed the questionnaire, thus representing a nearly 5% response rate. Although not statistically significant, the results represent trends in practice that were further discussed during meetings with the Good CME Practice Group Spring Meeting (London, May 7, 2015) and the EBAC Meeting with Providers and Sponsors (Frankfurt, May 29, 2015). Below is an overview of each question, with all results being summarised at the conclusion.

## General Q1 – Q9

## Q1 Which best describes your organisation?

Answered: 54; Skipped: 1 [Table T0001]

**Table T0001:** 

	**Response percent**	**Response count**

Medical scientific society/association	14.8	8
Medical institution (hospital, clinic, university, etc.)	35.2	19
Individual physician or physician group	7.4	4
Event planning company/PCO (specialising in logistical organisation)	13.0	7
Company specialising in CME/CPD	16.7	9
Communications/ad agency	1.9	1
Journal	0.0	0
Industry (pharmaceutical, device)	3.7	2
Other (please describe)	7.4	4
• Publishing company • Specialty accreditation board • We are a PCO responsible for all congress organisation services except the scientific content of the congresses • We work on behalf of the university hospital and help them with the administrative tasks of a CME activity		


## Q2 Which best describes your organisation's role in providing CME/CPD activities?

Multiple answers possible; Answered: 52; Skipped: 3

Twenty-nine respondents at 55% said they organise everything themselves, of which 10 indicated that in some circumstances they also collaborate. Therefore, 19 of the 52 respondents, or 36%, are full-service providers organising all elements without external support. At 64%, the majority of respondents collaborate with other organisations.

**Figure:**
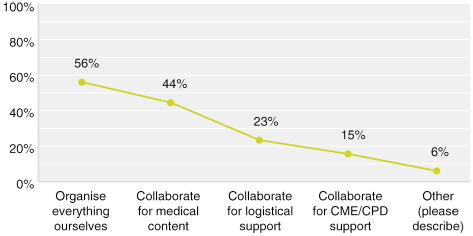


## Q3 Approximately how many years has your organisation been involved in CME/CPD?

## Q4 Approximately how many years have you personally been involved in CME/CPD?

Answered: 52; Skipped: 3

Organisations (Q3): 50% of the organisations responding to the survey were well established with over 10 years of experience, whereas 32% had less than 5 years history in CME/CPD. Individuals (Q4): indicating a similar but not identical trend as Q3, 37% of the individuals had over 10 years of experience, with 42% having under 5 years in CME/CPD.

**Figure:**
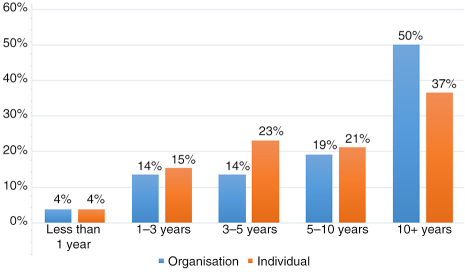


## Q5 What types of CME/CPD activities do you organise?

Multiple answers possible; Answered: 52; Skipped: 3

The vast majority of respondents, or 85%, organise live events for 0–500 participants. In addition to live events, 37% provide e-learning, 13% provide print/journal, and 4% CD-ROMs. One respondent said they also provide exams and another said they provide workshops and seminars.

**Figure:**
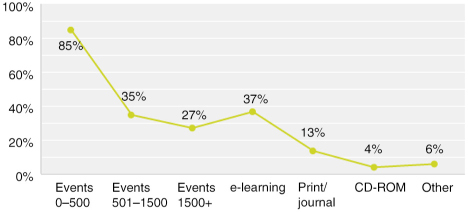


## Q6 In which geographic locations do you offer CME/CPD activities?

Multiple answers possible; Answered: 51; Skipped: 4

The most prominent response had 51% of respondents indicating they offer CME/CPD internationally in Europe. Most of those respondents marked multiple answers. Single responses to Q6 were:

- 7 (14%) organise only regionally in their local area
- 8 (16%) checked only nationally in their country
- 8 (16%) checked only internationally in Europe
- 6 (12%) checked only internationally globally

## Q7 From which organisations have you received CME/CPD credit in the past 5 years?

Multiple answers possible; Answered: 51; Skipped: 4

Naturally, given the target audience, EBAC had the most responses. However, surprisingly, 14 of the 51 respondents had not received CME/CPD credit from EBAC over the previous 5 years. About half of the respondents checked more than one answer, of which the event planning companies and companies specialising in CME/CPD checked the most multiples.

**Figure:**
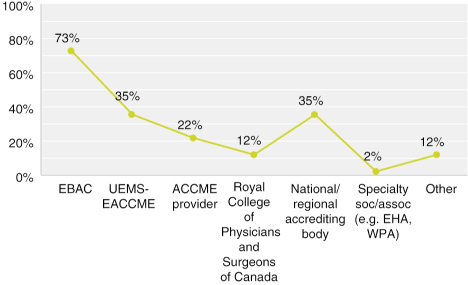


## Q8 Please indicate to what extent you agree or disagree with the following

Answered: 52; Skipped: 3 [Table T0002]

**Table T0002:** 

	**Strongly agree**		**Agree**		**Neutral/unsure**		**Disagree**		**Strongly disagree**	
									
CME/CPD accreditation adds no value.	3	6.0%	2	4.0%	8	16.0%	15	30.0%	22	44.0%
The resources necessary to obtain credit (cost, time, administration) outweigh the benefits of offering credits.	6	11.8%	13	25.5%	9	17.6%	17	33.3%	6	11.8%
CME/CPD accreditation is an indicator of high-quality education.	23	46.0%	15	30.0%	6	12.0%	5	10.0%	1	2.0%
CME/CPD credit is an important reason for participants to attend.	12	24.5%	21	42.9%	9	18.4%	6	12.2%	1	2.0%
CME/CPD accreditation ensures independent content free from industry influence and bias.	10	19.6%	26	51.0%	10	19.6%	2	3.9%	3	5.9%
We have considered organising medical education that does NOT offer CME/CPD credits.	6	12.0%	18	36.0%	12	24.0%	9	18.0%	5	10.0%

**Q8 by respondent group: CME/CPD accreditation adds no value**

Seventy-four percent (30 respondents) disagreed with the statement, thus expressing that there was strong agreement that accreditation does provide added value. However, the 26% (13 respondents) who were neutral or agree was not negligible and indicated a quarter of those actively involved in CME were not necessarily convinced of its value.

**Figure:**
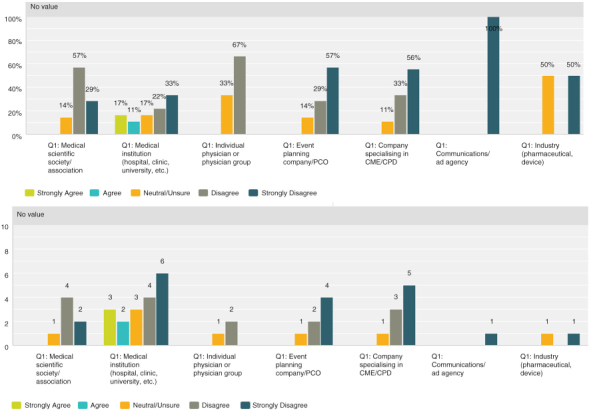


**Q8 by respondent group: The resources necessary to obtain credit outweigh the benefits of offering credits**

Overall, the responses were closely split with 37% agreeing (19 respondents) and 45% (23 respondents) disagreeing. A breakdown by provider type illustrates that medical institutions and physicians tend to find that the resources necessary do indeed outweigh the benefits. While organisations who specialise in organising the logistical or CME/CPD elements disagreed and found the resources required worth the benefits of offering credits.

**Figure:**
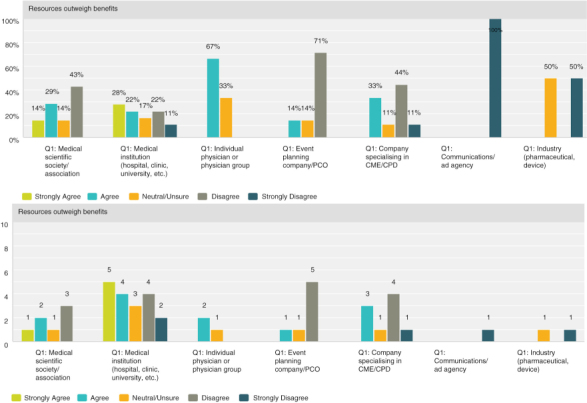


**Q8 by respondent group: CME/CPD accreditation is an indicator of high-quality education**

Seventy-six percent (38 respondents) agreed with the statement while the remaining 24% (12 respondents) were split evenly between neutral/unsure and disagreement. The event planning companies and companies specialising in CME/CPD tended to agree while the most varied answers were from the medical institution group.

**Figure:**
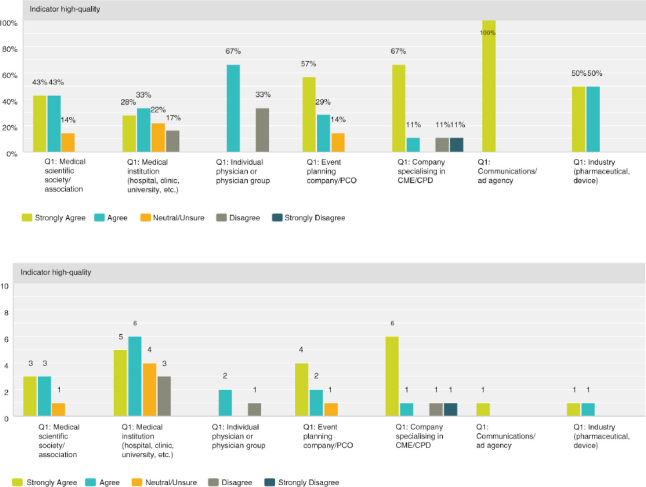


**Figure:**
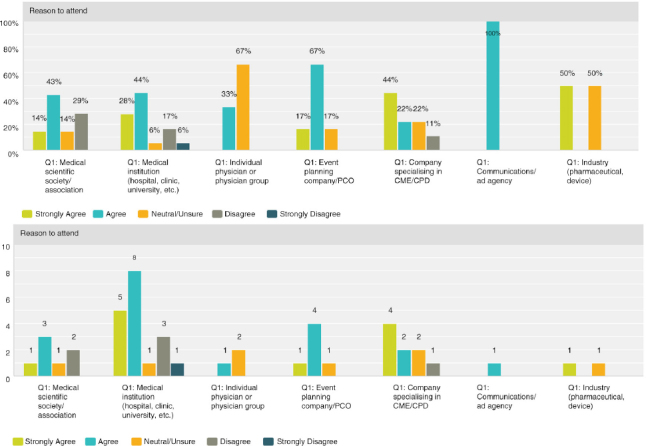


**Q8 by respondent group: CME/CPD is an important reason for participants to attend**

Overall, 67% (33 respondents) agreed with the statement, with 72% (13 respondents) of medical institutions and 84% (5 respondents) of event planning companies agreeing most strongly.

**Figure:**
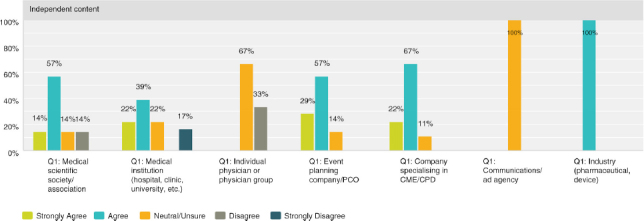


**Q8 by respondent group: CME/CPD accreditation ensures independent content free from industry influence and bias**

The role of accreditation in ensuring independence from bias was validated overall with 70% (36 respondents) agreeing and only 10% (5 respondents) disagreeing. However, a significant 20% (10 respondents) was unsure. Thirty-three percent of physicians (1 respondent) outright disagreed and 67% (2 respondents) were neutral or unsure.

**Figure:**
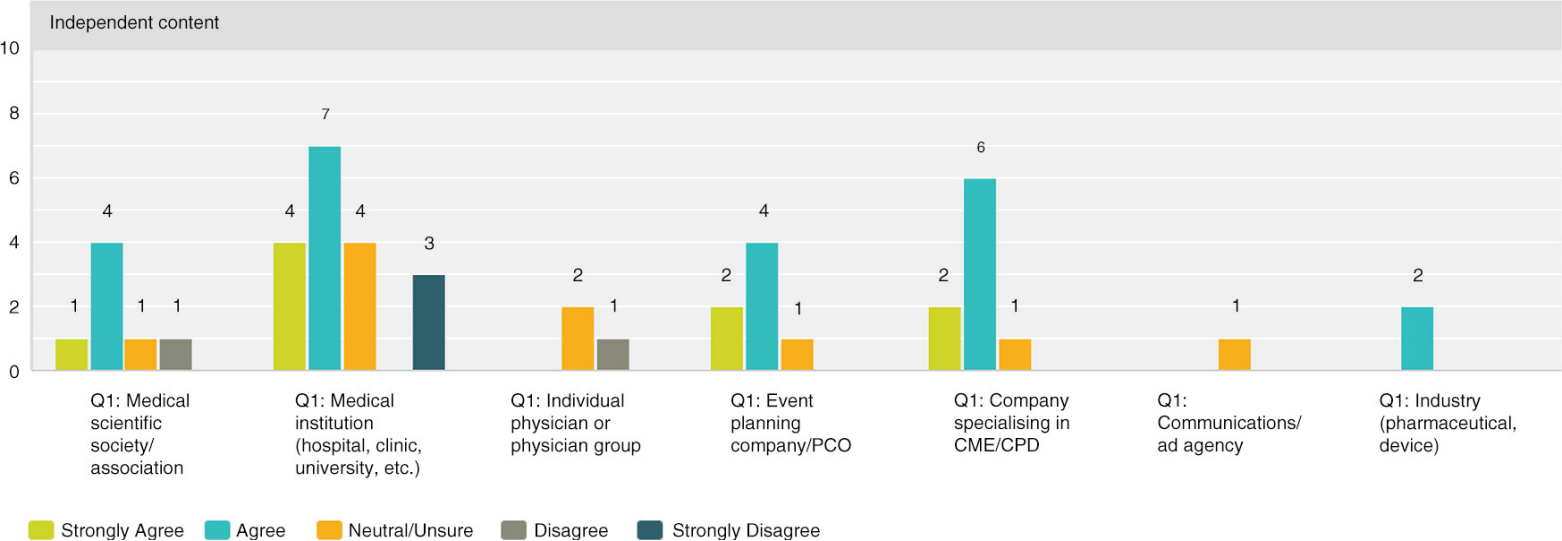


**Figure:**
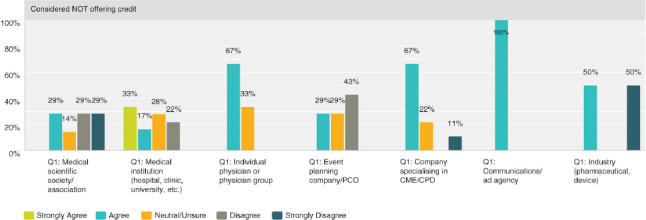


**Q8 by respondent group: We have considered organising medical education that does NOT offer CME/CPD credits**

Overall 48% (24 respondents) agreed with the statement while 28% (14 respondents) disagreed, with those answers of disagreement coming primarily from medical institutions at 58% (4 respondents) and medical societies/associations at 22% (4 respondents).

**Figure:**
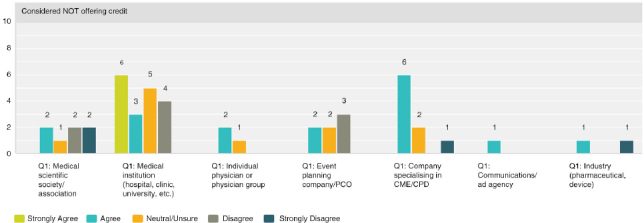


## Q9 Overall, in your personal opinion, do you think the importance of CME/CPD accreditation will: increase, decrease, stay the same, you're not sure

Answered: 53; Skipped: 2

Sixty-two percent believed the importance of CME/CPD accreditation would increase, while 25% thought it would stay the same, 8% thought it would decrease, with 6% unsure. Most of the decrease answers came from physicians and medical institutions.

## Implementing the Accreditation Criteria Q10 – Q18

## Q10 Ideally, in your opinion, who should be responsible for the following?

Answered: 48; Skipped: 7 [Table T0003]

**Table T0003:** 

	**Director, chair, committee**		**Org. team**		**Unsure/no opinion**	
					
Content/topic selection	42	87.5%	6	12.5%	0	0.0%
Faculty/speaker selection	39	81.3%	8	16.7%	1	2.1%
Educational format selection	21	43.8%	26	54.2%	1	2.1%
Content/presentation review prior to event	33	68.8%	12	25.0%	3	6.3%
Creation of educational materials	8	16.7%	36	75.0%	4	8.3%
Collection/management of all conflicts of interest declarations/disclosures	10	20.8%	36	75.0%	2	4.2%
Evaluation	12	25.0%	34	70.8%	2	4.2%
Management of industry funding and sponsorships	10	20.8%	38	79.2%	0	0.0%
Comments: • The content should be decided by physicians who are experts in the field. • Our organisation is doing all from a to z: physician, course director, and organisational team. • Content review (4) should be done by both the chair/session chair and the provider. • I am not sure what you mean by “presentation review prior to event.” • Content review must be made by physician with academic degree, independent of the faculty and recruited by EBAC or similar. • All scientific contents shall be handled by the scientific hosts; all logistical and administrative contents shall be handled by the PCO.						


## Q11 In reality, when actually planning a CME/CPD activity, who is most often responsible for the following?

Answered: 47; Skipped: 8 [Table T0004]

**Table T0004:** 

	**Director, chair, committee**		**Org. team**		**Unsure/no opinion**	
					
Content/topic selection	37	78.7%	8	17.0%	2	4.3%
Faculty/speaker selection	36	76.6%	9	19.1%	2	4.3%
Educational format selection	30	63.8%	14	29.8%	3	6.4%
Content/presentation review prior to event	32	68.1%	10	21.3%	5	10.6%
Creation of educational materials	15	31.9%	31	66.0%	1	2.1%
Collection/management of all conflicts of interest declarations/disclosures	16	34.0%	30	63.8%	1	2.1%
Evaluation	15	31.9%	30	63.8%	2	4.3%
Management of industry funding and sponsorships	13	28.9%	31	68.9%	1	2.2%
Comments: • As an accreditor, we do not necessarily know who actually does what in the organisation. • As a provider of CME/CPD, we make every effort to ease the faculty's resource burden. • Our organisation is doing all from a to z: physician, course director and organisational team. • We are lucky to be able to do what we think is best. Faculty (creators of content and reviewers) do not always engage in “best practice.” • Industries should be able to get EBAC accreditation for events planned by them.						


## Q10 and Q11 Comparison: Ideally versus Reality

Comparing responses for the following areas illustrates a parallel in what respondents feel should ideally be the task distribution and what is actually reportedly as happening.

**Figure:**
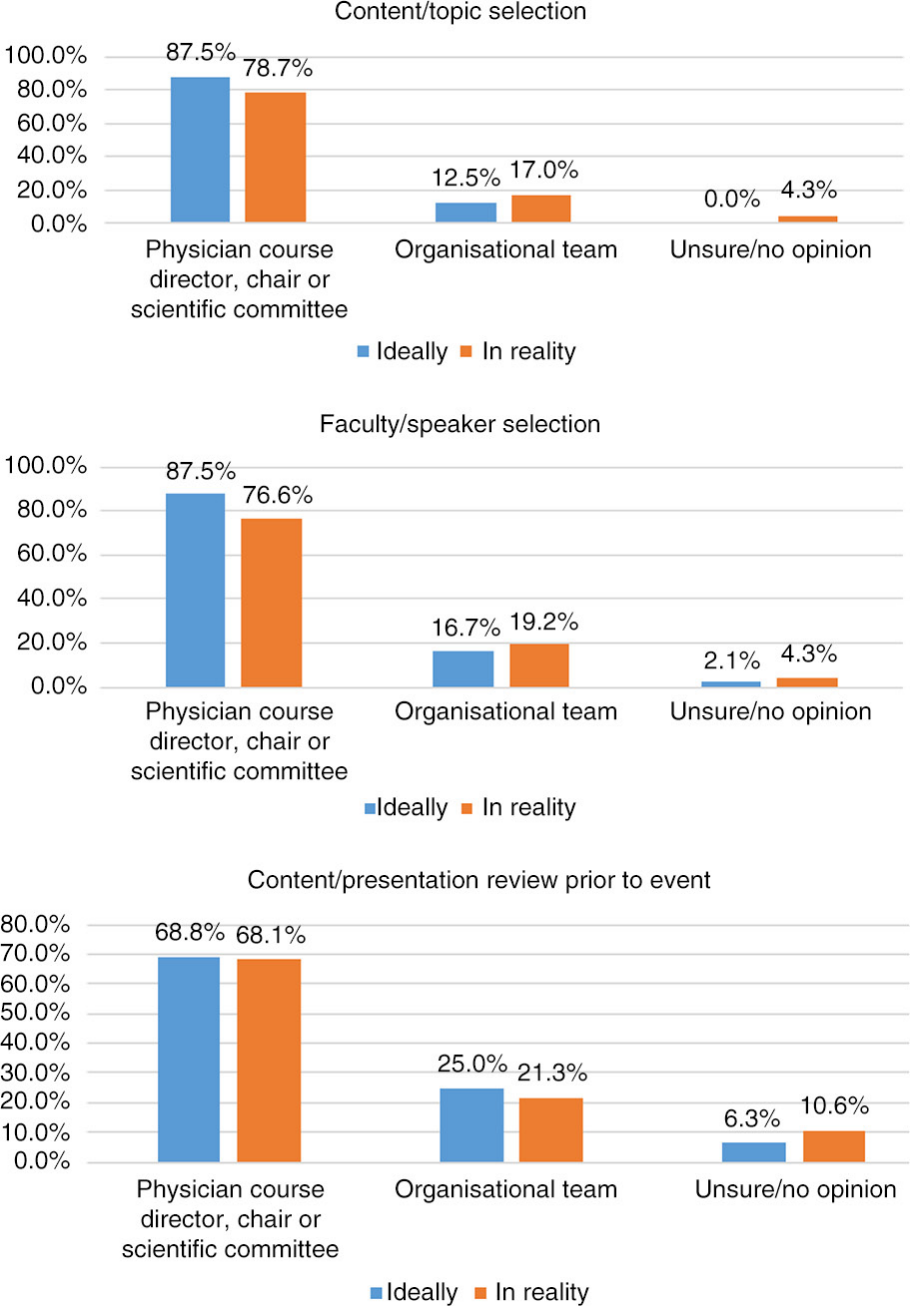


**Figure:**
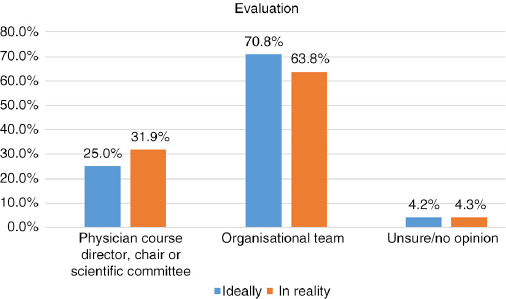


Similarly, when comparing the responses, the following areas reflected the most striking variances: educational format selection, creation of educational materials, collection/management of all conflicts of interest declarations/disclosures, and management of industry funding and sponsorships. For these areas, medical institutions perceived the physician course directors to be contributing most actively, while CME/CPD and event companies indicated willingness to play a more active role, indicating in almost every area “organisational team” for both ideal and reality. As such, the following may be areas for improving communication between parties and clearer distribution of roles and responsibilities.

**Figure:**
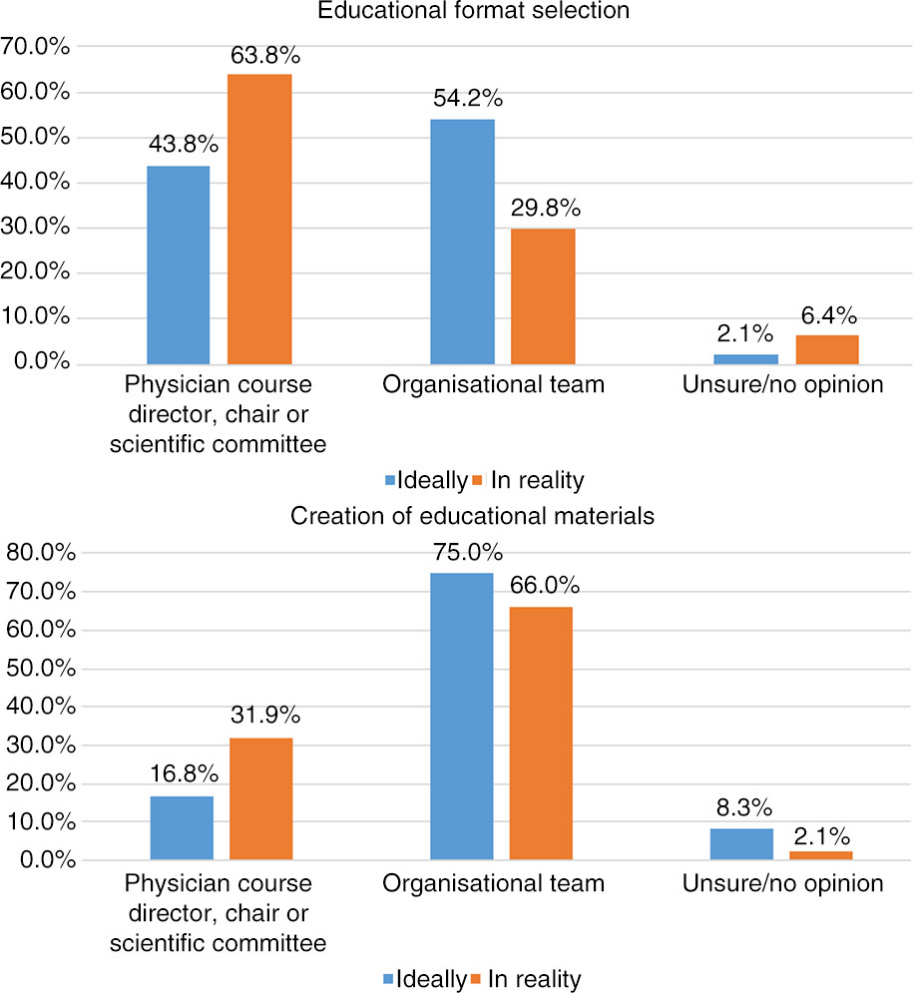


**Figure:**
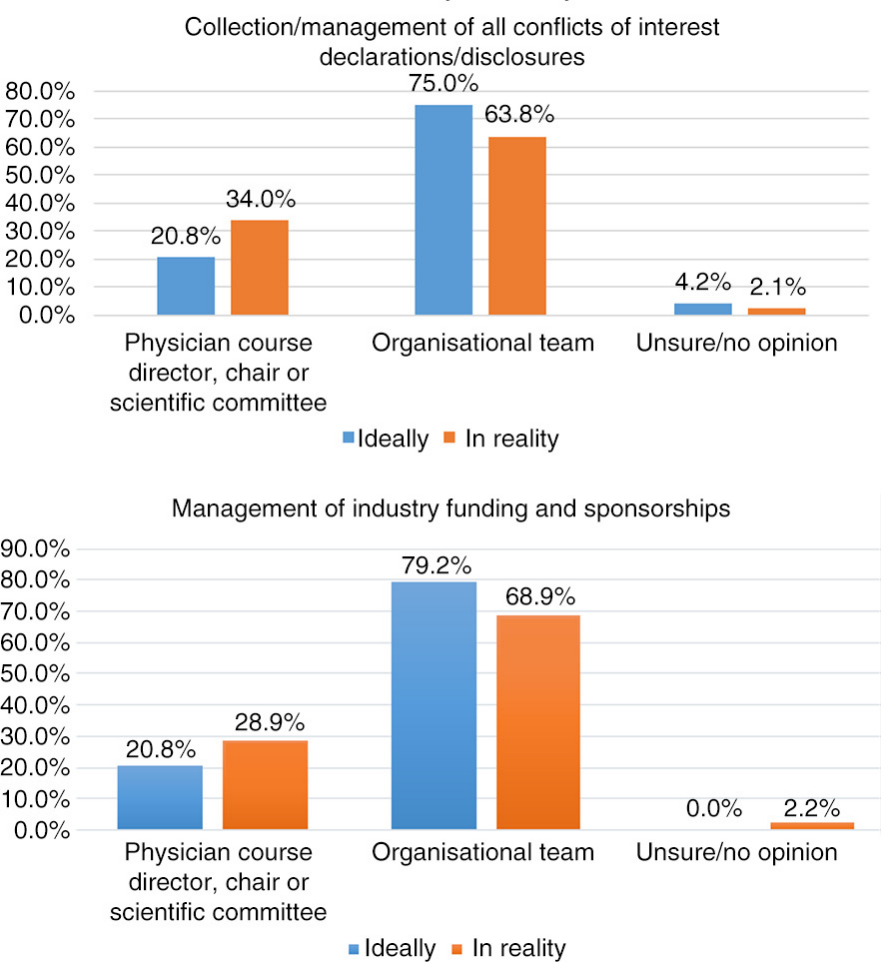


## Q12 Rank the following in terms of your ability to understand and apply to your CME/CPD activities

Answered: 46; Skipped: 9

Highest activities judged easy to extremely easy:

- Identifying a physician course director/chair (70%)
- Separating industry promotional activities (63%)
- Tracking attendance/credits and issuing certificates (58%)
- Creating educational objectives (57%)
- Understanding where advertisements can/cannot be located (57%)

Highest activities judged difficult to extremely difficult:

- Collecting all conflicts of interest disclosure forms from committees/faculty (30%)
- Performing an evaluation to assess educational outcomes (30%)
- Identifying professionals with CME/CPD expertise to assist (28%)
- Resolving any conflicts of interest (29%)
- Implementing interactive educational formats (26%)

Of note with high neutral/unsure responses:

1. Resolving any conflicts of interest (29%)
2. Performing a needs assessment (35%)
3. Understanding where industry sponsors can/cannot be involved (35%)

## Q13 How do you choose the content/topics for your CME/CPD activities?

Multiple answers possible; Answered: 47; Skipped: 8

**Figure:**
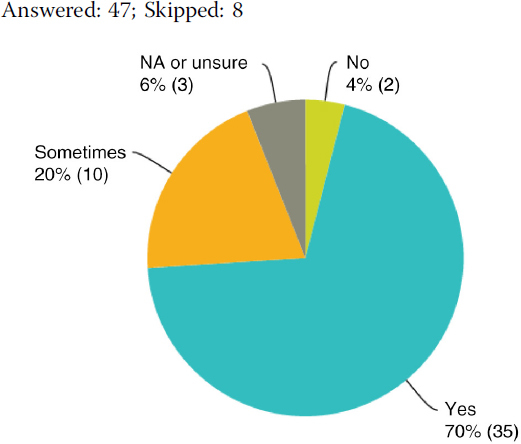


Most of the respondents use multiple methods with the top three answers being:

- 79% selected by physician course director or scientific committee
- 51% needs assessment done by participants of similar events
- 40% topics recommended by national authorities [Table T0005]

**Table T0005:** 

	**Response percent**	**Response count**

Needs assessment done by participants of similar events	51.1	24
Gap analysis by knowledge-based assessment	40.4	19
Gap analysis by assessment of individual performance	19.1	9
Number of participants in previous events	27.7	13
Topics recommended by national authorities	40.4	19
Topics selected by physician course director or scientific committee	78.7	37
Topics based on our own health-care system research	21.3	10
We are not involved in the process of choosing topics	12.8	6
What is a gap analysis?	8.5	4
What is a needs assessment?	2.1	1
Other (please describe)		4
• We do not perform needs assessments as we are the accreditor. • Outcomes from related activities. NA surveys of the target audience. • One addition: our starting point is a literature review. • Our scientific hosts choose the contents and topics based on their experience and knowledge they want to share.		


## Q14 Do you evaluate your CME/CPD activities?

Answered: 47; Skipped: 8

## Q15 Why don't you evaluate your CME/CPD activities?

No responses.

(Of those responding “yes” to Q14)

## Q16 Please indicate the evaluation method you use most often

Answered: 33; Skipped: 22

Fifty-eight percent do onsite evaluations, 18% do online evaluations, and 24% do both. Sixty percent use customised forms and 40% standardised forms. Medical institutions and societies lean on standardised forms more than companies specialising in CME/CPD and event planners.

**Figure:**
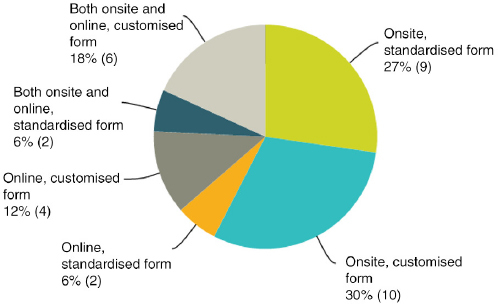


(Of those responding “yes” to Q14)

## Q17 For which purposes do you use the evaluation results?

Multiple answers possible; Answered: 35; Skipped: 20

The top three answers were: measure educational quality, selection of topics for future events, and feedback to presenters. Only 34% indicated they use evaluations to measure independence from commercial interests. Of which, respondents were mostly from companies specialising in CME/CPD. [Table T0006]

**Table T0006:** 

	**Response percent**	**Response count**

Feedback to presenters	65.7	23
Selection of presenters	51.4	18
Selection of topics for future events	71.4	25
Selection of educational formats for future events	62.9	22
Control of independence from commercial interests	34.3	12
Measure educational quality	74.3	26
We are not involved in the evaluation process	5.7	2
Other (please describe)	8.6	3
• Demonstrating return on investment to the sponsor. • The congress evaluation is summarised in a report, which we present to the Scientific Committee after the congress. This report serves as basis for the Scientific Committee to plan the next congress. • Also to feedback appropriate parts to the financial supporters.		


(Of those responding “sometimes” to Q14)

## Q18 Why do you only sometimes evaluate your CME/CPD activities?

Multiple answers possible; Answered: 10; Skipped: 45

**Figure:**
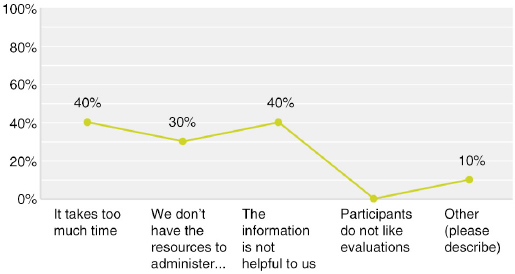


## EBAC Accreditation and Support Q19 – Q24

## Q19 For how many activities do you seek EBAC accreditation annually?

Answered: 45; Skipped: 10

**Figure:**
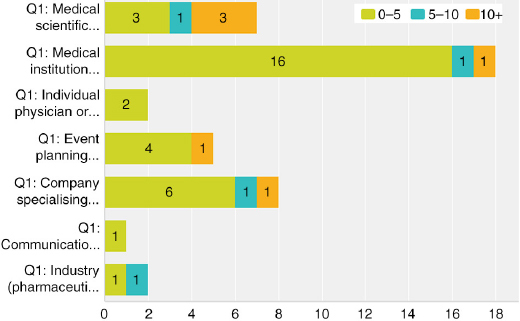


## Q20 Please indicate your satisfaction with the following aspects of EBAC accreditation

Answered: 45; Skipped: 10

The highest satisfaction scores (satisfied and very satisfied):

- Overall EBAC experience (71%)
- Clarity of the process, rules, and responsibilities (64%)
- Clarity of whom the official CME provider/applicant must be (64%)
- Requirement to have a Letter of Endorsement (61%)

The highest unsatisfied scores (unsatisfied and very unsatisfied):

- Requirement that each event must be submitted separately, even if a recurring/repeating (16%)
- Clarity of whom the official CME provider/applicant must be (13%)
- Requirement to have a Letter of Endorsement (14%)
- 8 week minimum application deadline (11%)

For all the categories, the majority of respondents were satisfied (and more satisfied than very satisfied). Each category also had a strong showing of neutral/unsure answers ranging from 22% to 38%.

**Figure:**
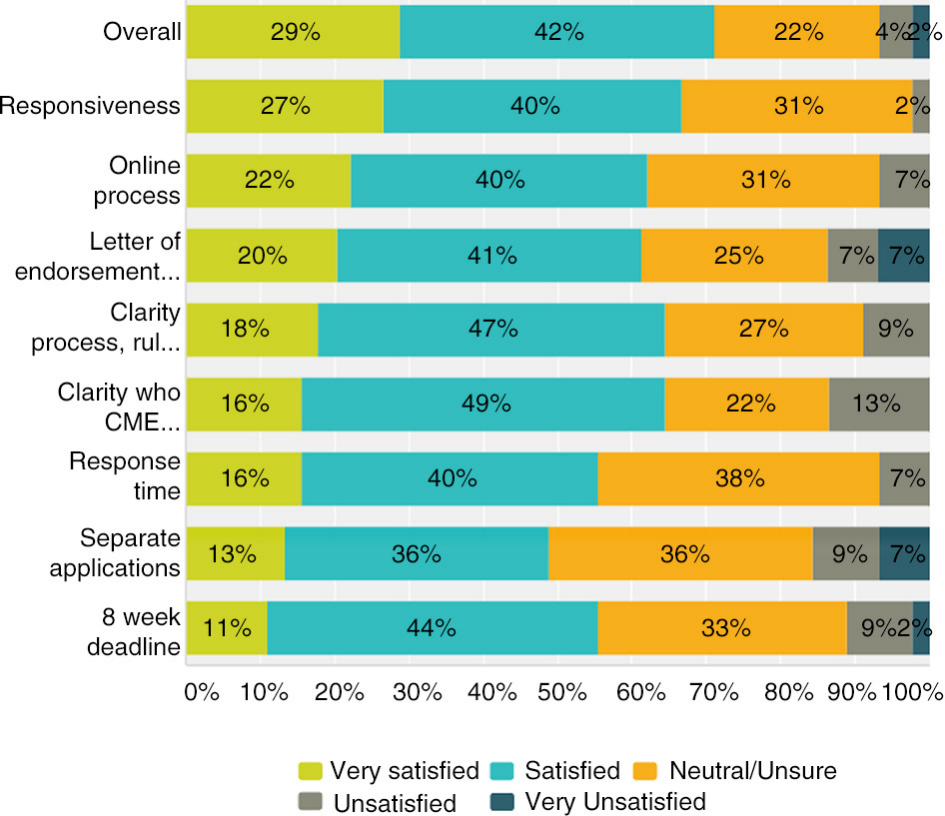


## Q21 What would you recommend to CME/CPD accreditors like EBAC?

Multiple answers possible; Answered: 41; Skipped: 14 [Table T0007]

**Table T0007:** 

	**Response percent**	**Response count**

Less bureaucracy	43.9	18
Insist on needs assessment	22.0	9
Reject all industry-funded CME/CPD	2.4	1
Facilitate provider accreditation	36.6	15
Focus on e-learning and fewer points for live meetings	14.6	6
Focus on live meetings and fewer points for e-learning	19.5	8
Strengthen the role of commercial sponsors	17.1	7
I'm not sure	17.1	7
Other (please describe)		8
• Online CME for less than 1 hour. • Educate the providers and faculty on what is/how to do a needs assessment and evaluating outcomes. Give guidance on how to develop good-quality education. Share data. • More tight relation with the faculty/lecturers in order to facilitate and speed up the review process of lecturers and content/presentations. • Lower the fee – unfortunately some events we organised in the past could not afford the accreditation fee and therefore were not accredited by EBAC; this is a sore point as most of EU participants do require EBAC accreditation. • Decrease price. • System more user friendly. • Provide recommendation on best practice for needs assessment and evaluation. • The only thing I can think of is concerning the submission of the final report. EBAC requires the final report to be submitted within 4 weeks after the CME activity has taken place, could this be changed to 6 weeks?		


## Q22 If you could instantly change one thing about the process for obtaining CME/CPD credits, what would it be and why?

Answered: 18; Skipped: 37

Responses are as written by respondents. Most answers focused on the process of obtaining credits: applications, timelines, expenses (“price is too high for added value”), and bureaucracy (“make it easier to apply”).

- One application for recurring events with single annual fee.
- Simplify reaccreditation of an already conducted activity.
- Make sure that one application is submitted which will also be able to include sessions supported by grants.
- Grant it online to those who attend.
- More tight relation with the faculty/lecturers in order to facilitate and speed up the review process of lecturers and content/presentations.
- Lately the decision from EBAC is taking a longer time, it could be due the increased number of applications received, ideally 5 weeks would be great, it would facilitate the planning process, there are many other deadlines to meet while organising a CME event, so that would be ideal.
- The time to obtain the credits: more transparency and compliance with deadlines as to launch a CME programme. The process should be easier because we are in a very fast-moving sector, and we should be able to have a CME activity in max. 1 month, but it is not possible because we first have to get everything ready as if we launched the programme, then send to have the credits and wait a lot to obtain them. There should be other ways to credit the CME providers and have a self-code of conduct with controls, but this would allow the CME provider to be more effective and the doctors to have CME faster.
- It would be great if the process could be split up in two steps. Step 1: As soon as destination is announced, we can apply for CME credits with no programme; we just get a positive or negative reply from CME provider (no specific number of credits), but we still get to use their logo and say that the event will be EBAC accredited (EBAC can have an option to pull out at step 2). Step 2: As soon as advance programme is available, we make the full application and EBAC give us the number of credits.
- Most independent reviewers we have worked with (anonymously through RCP and EACCME) have demonstrated a clear lack of understanding about e-learning formats yet insist on making most comments about the delivery of the education, whereas ACCME-accredited providers who collaborate with CME providers tend to focus on ensuring the criteria are followed (conflicts of interest, needs assessments, learning objectives, outcomes) and allow the CME providers to identify the ideal delivery and coordinate the faculty and content development.
- To have a better control on attendance, so not everyone can get CME/CPD credits! We/you should create a system (electronic maybe) to know how many hours the attendees were present at the seminars.”
- Instantly forbid any medical communications agency from being involved in CME and clarify a grant process that is EFPIA compliant. Everyone wants clarity that industry is not involved in CME, and these two things would give it instantaneously.

## Q23 Do you have other questions or comments? Don't be shy, let us know

Answered: 13; Skipped: 42

- Assessment should be based on knowledge and courses attendance.
- CME credits overall just meet an administrative need and do not reflect knowledge/experience gained.
  If the CME credits requirements are waived, the folks may attend meetings with the idea of getting knowledge and not just to get credits. I do think it is important to stay up to date with science but not sure CME collected in the current format is the correct way to track the activity. Not sure what will be the right process do so.
- This survey is not exactly relevant for us as accreditors.
- CME should be from practical points and good illustrated.
- CME/CPD means so many different things around the world that it is difficult to justify online CME/CPD for a global audience.
- Currently the number of credits corresponds to the number of hours a delegate could attend whatever the subject or way of learning; why would someone that watches a webinar online for 1 hour get the same points as someone that attends a 1-hour “how to” training session in person with a world-renowned expert.
- Let industries get EBAC credit for events organised by industries.
- Nice survey Julie. I hope that the results will be published in *JECME*.
- No.
- Overcome double accreditation with EBAC and national/international societies.
- Thanks!
- The latest EBAC evaluation form is extremely long with too many questions. Doing big international congresses, participants attending a 1-hour session have no time to complete this, they have to run to next session, so it is really unfortunate that we are not able to collect many of those forms, evaluating a session is extremely useful for all of us.
- The need for independent education for physicians with accreditation and CME will increase even for small events and the borders between sponsors and professions must be transparent – this implicates development of methods and the process for accreditation.

## Q24 Would you be willing to provide additional input during a short telephone call?

11 respondents submitted their email address. [Table T0008]

**Table T0008:** 

	**Response percent**	**Response count**	

Medical scientific society/association	0	0	
Medical institution (hospital, clinic, university, etc.)	45	5	UK (3), Sweden (1), India (1)
Individual physician or physician group	9	1	USA (1)
Event planning company/PCO (specialising in logistical organisation)	18	2	Israel (1), Netherlands (1)
Company specialising in CME/CPD	18	2	Spain (1), UK (1)
Communications/ad agency	0	0	
Journal	0	0	
Industry (pharmaceutical, device)	9	1	France (1)

## Summary

The survey respondents represented a relatively broad range of provider types with only journal going unrepresented and medical institutions most prominent at 19 (Q1). The majority of providers collaborate with other organisations for various aspects of the activities, although 36% are “full-service” providers managing all elements directly (Q2). Overall, the group was a relatively knowledgeable one with many years’ experience in CME/CPD both organisationally and individually (Q3, Q4). Live events are the primary offering across all groups (Q5), with such events being offered regionally, nationally, and internationally (Q6). As such, respondents are obtaining credits from a range of accrediting bodies (Q7).

Though results of this survey cannot be considered as representative in any of the subgroups, they nevertheless provide recent data from the field and demonstrate opinions often encountered by EBAC in current discussions on CME/CPD accreditation. Further, although the majority of respondents recognised the value of CME/CPD accreditation, they also expressed that they have considered offering activities without credits at all (Q8). Moreover, the accreditation procedure itself has been embarrassingly considered as at least potentially:

- a purely bureaucratic exercise with investment of resources outweighing the benefit (62.7%)
- not ensuring educational quality (24%) or
- independence of information (29.4%), and thus
- adding no value (26%) (Q8).

However, 87% agreed that the importance of CME/CPD accreditation will at least remain the same, with the likelihood that it will actually increase (Q9).

Responses also demonstrated clearly that planning and delivery of CME/CPD is decidedly a team effort, with physician course directors and committees no longer directly managing all aspects (Q10, Q11). There were no surprises in the distribution of roles and responsibilities amongst these physician groups and supporting organisational teams. There was, however, some discrepancy between responsibilities in theory and in reality in regard to educational formats, educational materials, conflict of interest management, and management of industry funding, with the physician leads playing a more active role than what is considered ideal (comparison of Q10 and Q11). Correspondingly, respondents expressed a level of difficulty with these same areas while also indicating some difficulty with implementing an evaluation and appropriate outcomes measurement (Q12). Based on the results of the survey, the physician course directors, chairs, and committees are still the primary group responsible for selecting topics (79%), although most respondents indicated they use multiple methods for creating the programme content (Q13). This means that evaluation results are used for providing feedback to presenters, but also drive the design and content of future events (Q17).

These answers, in conjunction with the finding that 30% of respondents do not (or only occasionally) evaluate their CPD activities (Q14) and that a surprisingly high percentage of respondents do not see the medical professionals in a leading role when it comes to managing industry funding and conflicts of interest (Q10/11), may lead to the conclusion that concept and practice of CME/CPD accreditation need further promotion and dissemination in order to be understood by all stakeholders. This is even more important since the bulk of respondents only submit 0–5 applications annually to EBAC (76%) (Q19) and many providers expressed that CME/CPD represents only a portion of their job responsibilities. As such, most comments focused on ways to facilitate the integration of the accreditation requirements into operations, which are typically more dynamic than the accreditation process allows for (Q21–23). This includes transparency around the accreditation process itself and its ensuing credit decisions, as well as bridging the gap between the accreditation criteria in theory, the “what” to do, and the practical implementation of these standards into business realities, the “how” to do it. This manifests itself by being accessible for questions, providing education on key concepts, and offering concrete support in the form of tools, templates, and best practices.

In this regard, EBAC is satisfied with feedback regarding the accreditation process itself (Q19–21). For all the categories, the majority of respondents were satisfied with EBAC. Interestingly, and worth further exploring, each category also had a strong showing of neutral/unsure answers ranging from 22% to 38%. There are areas for improvement that were identified, namely around the applications process and timelines (Q20). This was also reflected in the recommendations that would be made to accreditors in general, which included less bureaucracy (44%) and facilitation of provider accreditation (37%) (Q21).

Though fully recognising the non-representative nature of the results, they nevertheless are consistent with the observations that EBAC reviewers have regularly made in the process of CME/CPD accreditation, as reflected in their comments on individual applications.

Thus, EBAC considers this survey as part of a continuing quality improvement process aiming to help providers and health-care professionals see CME/CPD accreditation as more than a tedious bureaucratic duty, but rather to regard it as an important contributor towards the delivery of highest possible standard of patient care.

